# Data on the effect of boiling on the organosulfides and the hydrogen sulfide-releasing activity of garlic

**DOI:** 10.1016/j.dib.2016.11.074

**Published:** 2016-11-24

**Authors:** Restituto Tocmo, Yuchen Wu, Dong Liang, Vincenzo Fogliano, Dejian Huang

**Affiliations:** a Food Science and Technology Programme, Department of Chemistry, National University of Singapore, 117543 Singapore; b Food Quality Design Group, Wageningen University, PO Box 8129, Wageningen, Netherlands; c National University of Singapore (Suzhou) Research Institute, 377 Lin Quan Street, Suzhou Industrial Park, Jiangsu 215123, China

## Abstract

This article contains experimental data on the identification and quantification of the organosulfides on boiled garlic extracts. Data included are related to the research article “*Boiling enriches the linear polysulfides and the hydrogen sulfide-releasing activity of garlic*” (R. Tocmo, Y. Wu, D. Liang, V. Fogliano, D. Huang, 2016) [1]. Characterization was carried out by GC–MS and HPLC. Dose-response curves obtained from the cell-based H_2_S-releasing capacity assay of allicin transformation products, namely vinyl dithiins and ajoene are also included. DATS-E values were calculated from these dose-response curves to quantify the contribution of the individual polysulfides to the H_2_S-releasing capacity of boiled extracts.

**Specifications Table**

**Table:** 

Subject area	*Chemistry*
More specific subject area	*Food Chemistry*
Type of data	*Table and figure*
How data was acquired	*GC–MS (Agilent 7890A with an Agilent 5975C MS unit), HPLC (Waters HPLC system equipped with a Waters 2996 Photodiode Array Detector and a Waters 2695 Separation Modules, microplate reader (Synergy HT microplate reader)*
Data format	*Raw and Analyzed*
Experimental factors	*Garlic samples were boiled at different boiling times*
Experimental features	*Garlic was boiled at different boiling times and organosulfur compounds were analyzed by HPLC and GC–MS. H*_*2*_*S-releasing capacity of garlic extracts were quantified in parallel by a cell-based fluorescent H*_*2*_*S assay.*
Data source location	*Food Science and Technology Programme, Department of Chemistry, National University of Singapore, Singapore.*
Data accessibility	*Data are presented with this article.*

**Value of the data**

• GC–MS data provide identities, chemical structures, and mass spectral features of the organosulfides in boiled garlic extracts.
• HPLC data give information on the organosulfide components of the same boiled garlic extracts analyzed by a method that does not expose the extracts to high temperatures.
• The representative dose-response curves provide information on the quantification of H_2_S-releasing activity of pure forms of major allicin transformation products, including isomeric vinyl dithiins and ajoene.

## 1 Data

**Fig. 1:**
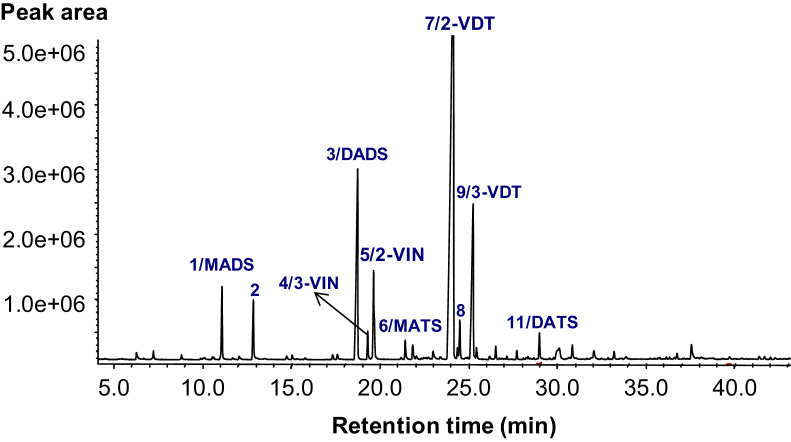
Representative GC chromatogram of polysulfides in boiled garlic extracts. Peak numbers correspond to the peaks listed in the first column of Table 1. The analytical conditions are described in the Materials and Methods [1]. 3-VIN: 3-vinyl-1,2-dithiane; 2-VIN: 2-vinyl-1,3-dithiane; 3-VDT: 3-vinyl-[4H]-1,2-dithiin; 2-VDT: 2-vinyl-[4H]-1,3-dithiin; MADS: methyl allyl disulfide; MATS: methyl allyl trisulfide; DADS: diallyl disulfide; DATS: diallyl trisulfide.

**Fig. 2:**
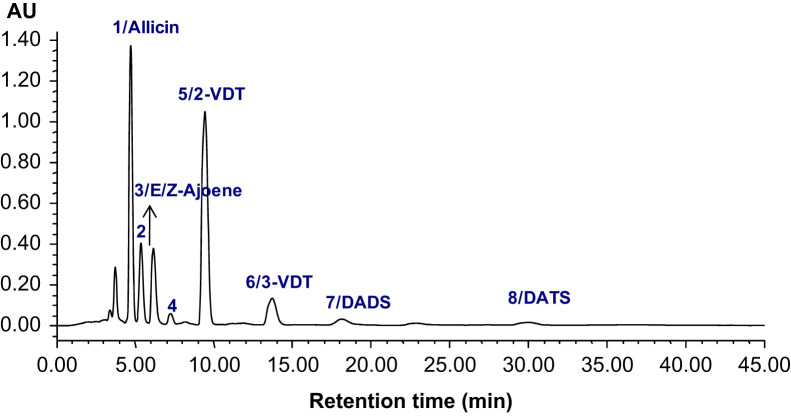
Representative HPLC chromatogram of polysulfides in boiled garlic extracts. The analytical conditions are described in Materials and Methods part [1].

**Fig. 3:**
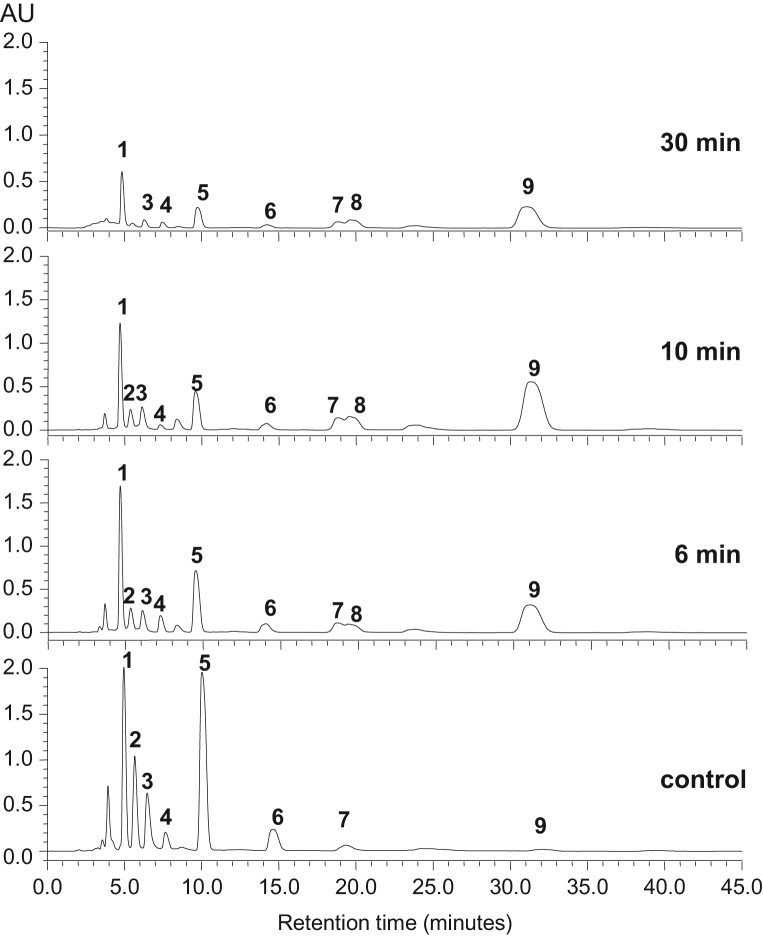
Representative chromatograms of garlic extracts after boiling. The analytical conditions are described in Materials and Methods part [1].

**Fig. 4:**
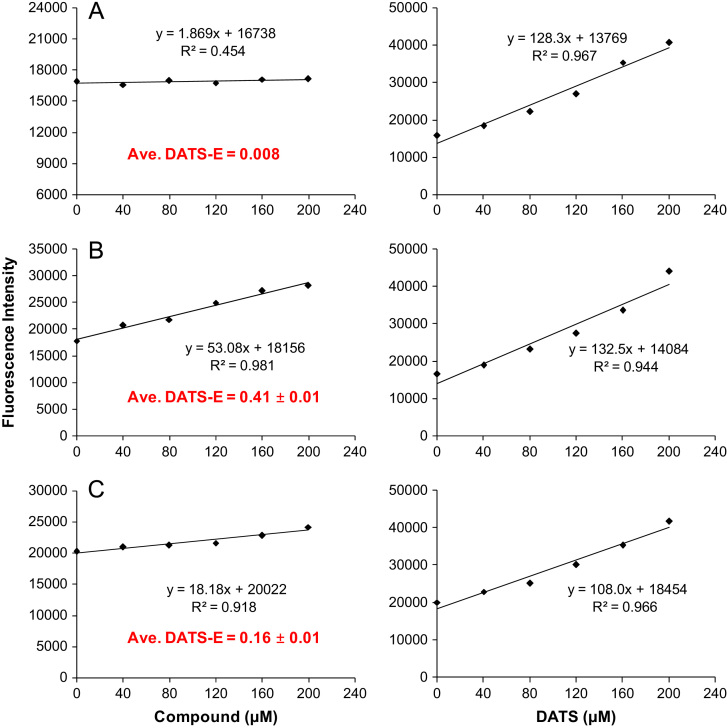
Representative curves of H_2_S-releasing assay for (a) 2-vinyl dithiin, (b) 3-vinyl dithiin, and (c) ajoene. Detailed description of the assay procedure is presented in Materials and Methods part [1].

The data presented include information on the organosulfur compounds in boiled garlic extracts as analyzed by GC–MS (Table 1 and Fig. 1) and HPLC (Fig. 2, Fig. 3). The allicin transformation products in garlic extract were isolated in pure forms and analyzed for their *in vitro* H_2_S-releasing activity (Fig. 4).

## 2 Experimental design, materials and methods

### 2.1 GC–MS analysis

Fresh garlic extracts were obtained by solvent extraction and their organosulfide constituents analyzed by GC–MS are presented in Table 1. The detailed descriptions of extraction procedure and mass spectral analysis are presented in the research article [1].

### 2.2 HPLC analysis of raw and boiled garlic extracts

The same garlic extracts (as described in Section 2.1) were analyzed by HPLC and a representative chromatogram is shown in Fig. 2. Complete description of the HPLC analysis is presented in the research article [1]. Furthermore, Fig. 3 shows representative HPLC chromatograms of solvent extracts obtained from garlic boiled at different times. The boiling treatment is described in the research article [1].

### 2.3 H_2_S-releasing capacity of allicin transformation products

Major allicin transformation products, including 2-vinyl dithiin, 3-vinyl dithiin, and ajoene were isolated by semi-preparative HPLC (described in detail in the research article [1]). Their H_2_S-releasing activity was measured using a cell-based (MCF-7 cells) H_2_S-releasing capacity assay employing an H_2_S selective and sensitive fluorescent probe. Representative dose-response curves obtained and the calculated DATS-E values are presented in Fig. 4. Detailed description of the *in vitro* H_2_S assay is presented in the research article [1] and in our previous published articles [2], [3].

## Figures and Tables

**Table 1:** Identity, chemical structures, and mass spectral data of the major organosulfur compounds in garlic.




^a^Refers to peaks in Fig. 1.

^b^Determined with Agilent 7890A quadruple mass spectrometer (Agilent, CA, USA), DB-5MS (30 m×0.25 mm ID, 0.25 μm film thickness) column, *m/z* with relative intensity in parenthesis, decreasing order. The analytical conditions are described in Materials and Methods part [1].

^c^Identification method: MS = mass spectrum compared to NIST 0.5a data base library; REF = identified my mass spectral data reported in the literature (Huang et al., 2010; Kimbaris et al., 2006; Sowbhagya et al., 2009; Yabuki et al., 2010).
